# A nomogram for predicting the cancer-specific death of children and adolescents-onset lymphoma: A SEER database analysis

**DOI:** 10.1097/MD.0000000000043781

**Published:** 2025-08-08

**Authors:** Jian Zhang, Mengqi Tai, Su’e Cao

**Affiliations:** a Department of Pediatrics, Affiliated Hospital of Weifang Medical University, Weifang City, Shandong Province, China.

**Keywords:** cancer-specific death, children and adolescents, competing risk, lymphoma, nomogram, prognosis, SEER

## Abstract

Lymphoma in children and adolescents represents a distinct clinical entity, often associated with aggressive biological behavior and poor cancer-specific outcomes. Accurate prediction of cancer-specific death (CSD) in this population is essential for guiding personalized treatment and follow-up strategies. This study aimed to develop and validate a nomogram for predicting the risk of CSD in children and adolescents with lymphoma using a competing risk model based on a large population-based cohort. Lymphoma cases diagnosed in patients aged 1 to 17 years were extracted from the surveillance, epidemiology, and end results database. Eligible patients were randomly assigned to a training cohort (70%) and a validation cohort (30%). Independent prognostic factors for CSD were identified using univariate and multivariate competing risk regression analyses. A nomogram was constructed based on significant variables, and its performance was evaluated by the concordance index (C-index), receiver operating characteristic curves, and calibration plots. A total of 6954 pediatric and adolescent lymphoma cases were included. Six variables, including age, race, diagnosis time, lymphoma subtype, tumor grade, and tumor stage, were identified as independent predictors of CSD. The nomogram showed strong discriminative power, with 5-, 10-, and 15-year area under curves of 0.814, 0.794, and 0.787 in the training cohort, 0.818, 0.792, and 0.764 in the validation cohort. Calibration curves demonstrated good agreement between predicted and observed outcomes. Survival analysis showed that patients with high-risk score had a poor clinical outcome. We developed a robust and clinically practical nomogram for predicting CSD in children and adolescents with lymphoma. This tool may assist clinicians in identifying high-risk patients and formulating individualized management strategies.

## 1. Introduction

Lymphoma is among the most common malignancies worldwide, with over 6,35,000 new cases and 2,73,000 deaths reported in 2022 alone.^[1]^ It encompasses a diverse group of hematological cancers, primarily classified into Hodgkin lymphoma and non-Hodgkin lymphoma, each with distinct biological behaviors and clinical outcomes.^[2]^ Lymphomas represent one of the most common malignancies in children and adolescents, accounting for approximately 15% to 20% of all childhood cancers.^[3,4]^ Molecular and pathological heterogeneity necessitates individualized risk stratification and treatment planning to optimize prognosis.

Children and adolescents group represents a unique clinical population due to distinctive disease biology, psychosocial challenges, and often more aggressive clinical behavior.^[5,6]^ Pediatric and adolescent-onset lymphomas frequently present at an advanced stage and carry a higher risk of recurrence, leading to poorer cancer-specific outcomes compared to adult-onset cases.^[7]^ Although the overall survival for pediatric lymphomas has improved with the advancement of multimodal therapy, cancer-specific death (CSD) remains a significant concern in long-term survivors.

Nomograms address these gaps by offering visual, quantitative tools that integrate diverse predictors to estimate personalized outcomes.^[8]^ They outperform traditional staging by providing continuous, patient-specific probabilities and enhancing clinical utility for shared decision-making. Although nomograms are established in adult oncology, their development and validation for CSD in pediatric/adolescent lymphoma populations are strikingly limited.

Current prognostic models for young lymphoma patients focus predominantly on overall survival or relapse risk, often neglecting cancer-specific mortality as an endpoint.^[9]^ This distinction is crucial, as competing risks (e.g., treatment-related sequelae, secondary malignancies) significantly confound survival analyses in this age group. Consequently, there is an urgent unmet need for a validated, pediatric-specific nomogram predicting CSD to guide risk-adapted therapy, improve resource allocation, and facilitate early intervention in high-risk subgroups.

This study aims to construct and validate the first nomogram for predicting cancer-specific mortality in children and adolescents with lymphoma using the data from the surveillance, epidemiology, and end results (SEER) database, leveraging comprehensive clinical, laboratory, and treatment-related variables. By addressing the unique epidemiologic and biologic context of this population, our model seeks to refine prognostic precision and ultimately contribute to personalized oncology care for young lymphoma patients.

## 2. Materials and methods

### 2.1. Data source and case selection

Data for this study were extracted from the SEER database, which covers approximately 26.5% of the U.S. population. SEER*Stat software (version 8.4.2) was used to identify lymphoma cases diagnosed in patients aged 1 to 17 years from 2000 to 2020. Inclusion criteria were as follows: histologically confirmed lymphoma; first primary malignancy; age at diagnosis between 1 and 17 years; complete follow-up information available; and cause of death attributed to lymphoma. Cases diagnosed solely by autopsy or death certificate, or with a survival time of <12 months, were excluded to minimize data inaccuracies.

Collected variables included age at diagnosis, sex, race, median household income, rural–urban residence, lymphoma subtype, tumor grade, tumor stage, radiotherapy, and chemotherapy status. Lymphoma histological subtypes were defined according to the International Classification of Diseases for Oncology, Third Edition (ICD-O-3). Ethical approval and informed consent were not required, as all data were de-identified and publicly available.

### 2.2. Statistical analysis

Patients were randomly assigned to either the training cohort (70%) or the validation cohort (30%). Comparisons between cohorts were conducted using the chi-squared test for categorical variables and Student *t* test for continuous variables. A competing risk model, as proposed by Austin et al,^[6]^ was applied to identify independent prognostic factors for CSD, with non-cancer deaths considered as competing events. This model is preferred over traditional Cox regression in the presence of competing risks, as it provides more accurate estimations of event probabilities.^[10]^

A predictive nomogram was constructed based on the independent risk factors identified in the training cohort using the “rms” and “survival” packages in R software (version 4.1.1). The model’s performance was evaluated using the C-index, receiver operating characteristic curves, and calibration plots. All statistical analyses were performed using SPSS version 25.0 (IBM Corp.) and R software, with a *P*-value <.05 considered statistically significant.

## 3. Result

### 3.1. Patient characteristics

**Figure 1.**
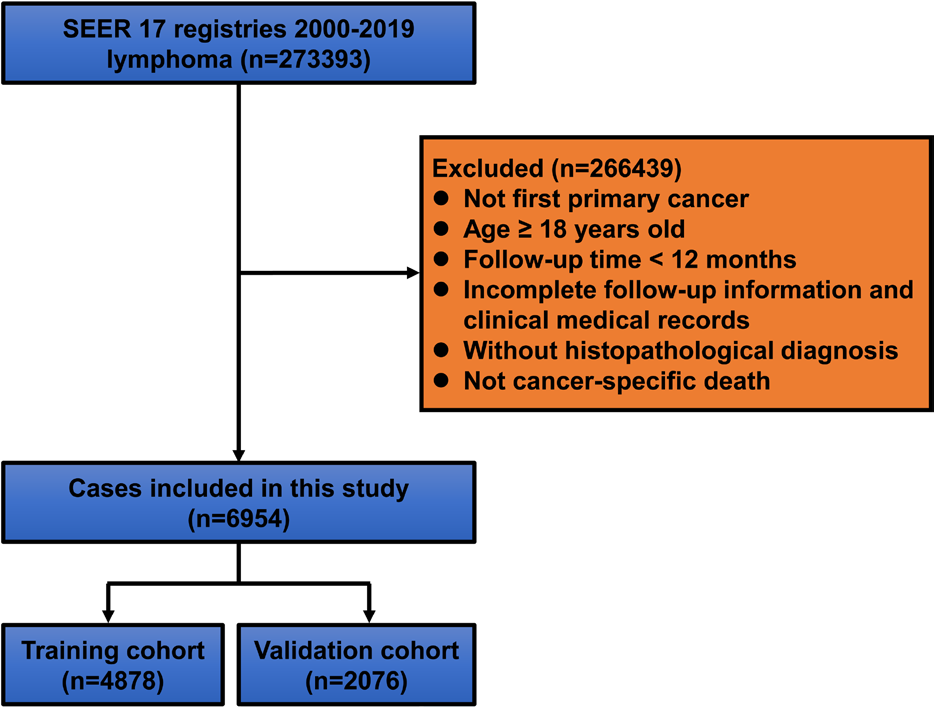
Study workflow. SEER = surveillance, epidemiology and end results.

Figure 1 showed the process of selected lymphoma cases. A total of 6954 eligible pediatric and adolescent lymphoma cases were identified and included in the analysis. These were randomly divided into a training cohort (n = 4878) and a validation cohort (n = 2076). Baseline characteristics of both cohorts are summarized in Table 1. The majority of patients were male (63%). Hodgkin lymphoma accounted for 47.8% (n = 3323) and non-Hodgkin lymphoma for 52.2% (n = 3631) of cases. Approximately 44% (n = 2995) of patients had distant-stage disease at diagnosis. About 49% (n = 3439) and 79% (n = 5497) of patients received radiotherapy and chemotherapy, respectively. There was no significant difference in CSD rates between the training and validation cohorts (Fig. S1, Supplemental Digital Content, https://links.lww.com/MD/P623).

**Table 1:** Clinical characteristics of lymphoma cases.

	Total cohort (n = 6954)	Training cohort (n = 4878)	Validation cohort (n = 2076)
Age	13 (9, 16)	13 (9, 16)	13 (9, 16)
Sex			
Female	2596 (37%)	1830 (38%)	766 (37%)
Male	4358 (63%)	3048 (62%)	1410 (63%)
Household income			
$75,000−	2783 (40%)	1983 (41%)	800 (39%)
$75,000+	4171 (60%)	2985 (59%)	1276 (61%)
Race			
White	5311 (76%)	3715 (76%)	1596 (77%)
Black	922 (13%)	646 (13%)	276 (13%)
Others	721 (11%)	517 (11%)	204 (9.8%)
Diagnosis time			
2000–2009	3479 (50%)	2438 (50%)	1056 (51%)
2010–2019	3475 (50%)	2440 (50%)	1020 (49%)
Rural–urban distribution			
Metropolitan area	6197 (89%)	4339 (89%)	1858 (89%)
Non-metropolitan area	757 (11%)	539 (11%)	218 (11%)
Subtype			
Hodgkin lymphoma	3323 (48%)	2317 (47%)	1006 (48%)
Non-Hodgkin lymphoma	3631 (52%)	2561 (53%)	1070 (52%)
Tumor grade			
B-cell	2355 (34%)	1651 (34%)	704 (34%)
T-cell	1097 (16%)	783 (16%)	314 (15%)
Others	3502 (50%)	2444 (50%)	1058 (51%)
Tumor stage			
Localized	1664 (24%)	1171 (24%)	493 (24%)
Regional	2295 (33%)	1594 (33%)	701 (34%)
Distant	2995 (43%)	2113 (43%)	882 (42%)
Radiotherapy			
No	3515 (51%)	2457 (50%)	1058 (51%)
Yes	3439 (49%)	2421 (50%)	1018 (49%)
Chemotherapy			
No	1457 (21%)	1024 (21%)	433 (21%)
Yes	5497 (79%)	3854 (79%)	1643 (79%)
Survival time	9.3 (4.8, 14.1)	9.4 (4.8, 14.2)	9.3 (4.9, 13.9)
Survival status			
Alive	6561 (94%)	4616 (95%)	1945 (94%)
Dead	393 (5.7%)	262 (5%)	131 (6%)

### 3.2. Construction and validation of the nomogram

**Figure 2.**
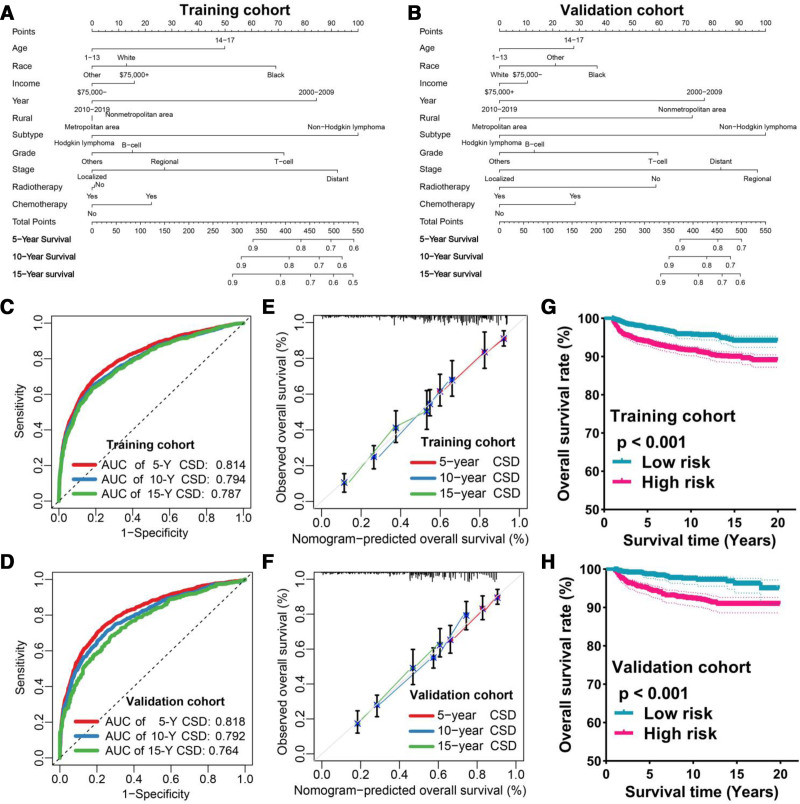
Construction and validation of the prediction nomogram. The nomogram for training (A) and validation (B) cohort. ROC curve (C, D), calibration curve (E, F), and survival curve (G, H) for training and validation cohort. AUC = areas under the curve, CSD = cancer-specific death, ROC = receiver operating characteristic.

Univariate and multivariate competing risk regression analyses identified 6 independent predictors of CSD: age, race, diagnosis time, lymphoma subtype, tumor grade, and tumor stage (Table 2; all *P* < .05). Based on these variables, we developed a prognostic nomogram to estimate 5-, 10-, and 15-year CSD probabilities (Fig. 2A for training cohort, Fig. 2B for validation cohort).

**Table 2:** The result of univariate and multivariate analysis.

	Univariate analysis		Multivariate analysis	
	HR (95% CI)	*P*-value	HR (95% CI)	*P*-value
Age				
1–13	Reference		Reference	
14–17	1.26 (1.03–1.55)	.024	1.49 (1.21–1.83)	<.001
Race				
White	Reference		Reference	
Black	1.62 (1.25–2.11)	<.001	1.60 (1.23–2.08)	<001
Other	1.27 (0.92–1.76)	.153	1.29 (0.93–1.78)	.132
Sex				
Female	Reference			
Male	1.05 (0.92–1.26)	.554		
Diagnosis time				
2000–2009	Reference		Reference	
2010–2019	0.58 (0.45–0.74)	<.001	0.53 (0.41–0.69)	<.001
Rural–urban distribution				
Metropolitan area	Reference			
Non-metropolitan area	1.13 (0.83–1.53)	.446		
Household income				
$75,000+	Reference			
<$75,000	1.11 (0.91–1.36)	.319		
Subtype				
Hodgkin lymphoma	Reference		Reference	
Non-Hodgkin lymphoma	1.59 (1.29–1.96)	<.001	1.59 (1.17–2.15)	.003
Tumor grade				
B-cell	Reference		Reference	
T-cell	1.74 (1.32–2.28)	<.001	1.56 (1.18–2.06)	.000
Others	0.90 (0.71–1.14)	.384	0.96 (0.70–1.31)	.805
Tumor stage				
Localized	Reference		Reference	
Regional	1.35 (0.99–1.83)	.054	1.63 (1.19–2.23)	.003
Distant	1.79 (1.35–2.38)	<.001	1.92 (1.44–2.56)	<.001
Radiotherapy				
No	Reference			
Yes	0.94 (0.77–1.15)	.562		
Chemotherapy				
No	Reference			
Yes	1.11 (0.88–1.41)	.381		

CI = confidence interval, HR = hazard ratio.

### 3.3. Model performance and clinical utility

The nomogram demonstrated good discriminative ability, with area under the receiver operating characteristic curves for the training cohort of 0.814 (5-year), 0.794 (10-year), and 0.787 (15-year), and for the validation cohort of 0.818, 0.792, and 0.764, respectively (Fig. 2C, D). The C-index values were 0.81 (95% confidence interval: 0.77–0.86) in the training cohort and 0.80 (95% confidence interval: 0.76–0.85) in the validation cohort.

Calibration plots revealed good agreement between predicted and observed outcomes in both cohorts (Fig. 2E, F). Kaplan–Meier analysis further demonstrated significantly worse outcomes for patients in the high-risk group based on nomogram scores (*P* < .001; Fig. 2G, H), indicating its potential for supporting individualized treatment decisions.

## 4. Discussion

In the present study, we developed and validated a novel competing risk nomogram to predict CSD in children and adolescents diagnosed with lymphoma, using a large population-based cohort from the SEER database. Unlike traditional Cox regression models that may overestimate cancer-specific risks by ignoring competing events,^[11,12]^ our nomogram integrates the cumulative incidence of CSD in the presence of other causes of death, providing a more accurate and clinically relevant prediction tool.

Our analysis identified several independent prognostic factors associated with CSD, including age at diagnosis, race, diagnosis time, lymphoma subtype, tumor grade, and disease stage. These findings are consistent with previous reports that have demonstrated the critical role of age and tumor biology in influencing lymphoma prognosis.^[13–15]^ Notably, older pediatric patients tended to have worse outcomes, which could reflect more aggressive tumor biology or decreased tolerance to intensive therapy.^[16,17]^ We also found that patients diagnosed in 2010 to 2019 had a significant lower risk of CSD compared with patients diagnosed in 2000 to 2009. One of the very important reasons is that he emergence of rituximab has improved the long-term prognosis of all patients with mature B-cell lymphoma.^[18]^ Moreover, BTK inhibitors and CAR T-cell therapy also changed the clinical outcome of lymphoma cases.^[19]^

Socioeconomic factors, particularly household income and rural–urban residence, are increasingly recognized as important determinants of CSD of lymphoma patients. Lower household income is often associated with delayed diagnosis, limited access to high-quality care, and suboptimal adherence to treatment protocols. In pediatric lymphoma, families with lower socioeconomic status may face barriers such as transportation challenges, inadequate health literacy, and higher rates of treatment abandonment, all of which contribute to poorer outcomes.^[20,21]^ Rural residence presents an additional layer of disparity. Children and adolescents living in rural areas may have limited access to specialized pediatric oncology centers, advanced diagnostics, and clinical trials.^[22]^ These limitations can lead to delays in diagnosis, less intensive therapy, or reduced access to hematopoietic stem cell transplantation, which is often necessary for high-risk lymphoma cases. Rural patients may also be more likely to receive care from generalists rather than oncology subspecialists, which can affect the quality and continuity of care.^[23]^

Racial disparities were also evident, with Black patients experiencing poorer outcomes compared to White patients, echoing findings in other malignancies such as breast,^[24]^ head and neck,^[25]^ and colorectal cancers.^[26]^ These disparities may reflect differences in access to care, socioeconomic factors, biological variations, or treatment compliance.

The nomogram demonstrated excellent discriminatory ability and calibration across both the training and validation cohorts. The area under curve values exceeded 0.8 for 5-, 10-, and 15-year CSD predictions, indicating high predictive performance. Additionally, survival analysis confirmed the clinical utility of the model, supporting its application in risk stratification and decision-making processes.

Nonetheless, several limitations should be acknowledged. First, despite the large sample size and rigorous methodology, selection bias and residual confounding cannot be entirely excluded. Second, certain potentially influential variables, such as genetic alterations, family history, treatment compliance, and socioeconomic status, were not available in the SEER database. Third, our findings are based on U.S. population data, which may limit generalizability to other healthcare settings or ethnic populations.

Despite these limitations, our nomogram provides a valuable, evidence-based tool to assist clinicians in predicting individualized CSD risk for children and adolescents with lymphoma. Its implementation may facilitate early identification of high-risk patients, enabling tailored therapeutic strategies and closer surveillance during long-term follow-up.

## 5. Conclusion

All in all, we developed a robust and clinically practical nomogram for predicting CSD in children and adolescents with lymphoma. This tool may assist clinicians in identifying high-risk patients and formulating individualized management strategies.

## Author contributions

**Investigation:** Jian Zhang.

**Methodology:** Mengqi Tai.

**Project administration:** Su’e Cao.

**Validation:** Mengqi Tai.

**Visualization:** Mengqi Tai.

**Writing** – **original draft:** Jian Zhang.

**Writing** – **review & editing:** Su’e Cao.

## Supplementary Material


