# Supplementary figures and images for: A nomogram for predicting the cancer-specific death of children and adolescents-onset lymphoma: A SEER database analysis
Source: Medicine (Baltimore). 2025 Aug 8;104(32):e43781. doi: 10.1097/MD.0000000000043781 (PMC12338206; doi:10.1097/MD.0000000000043781)

## Slide 1
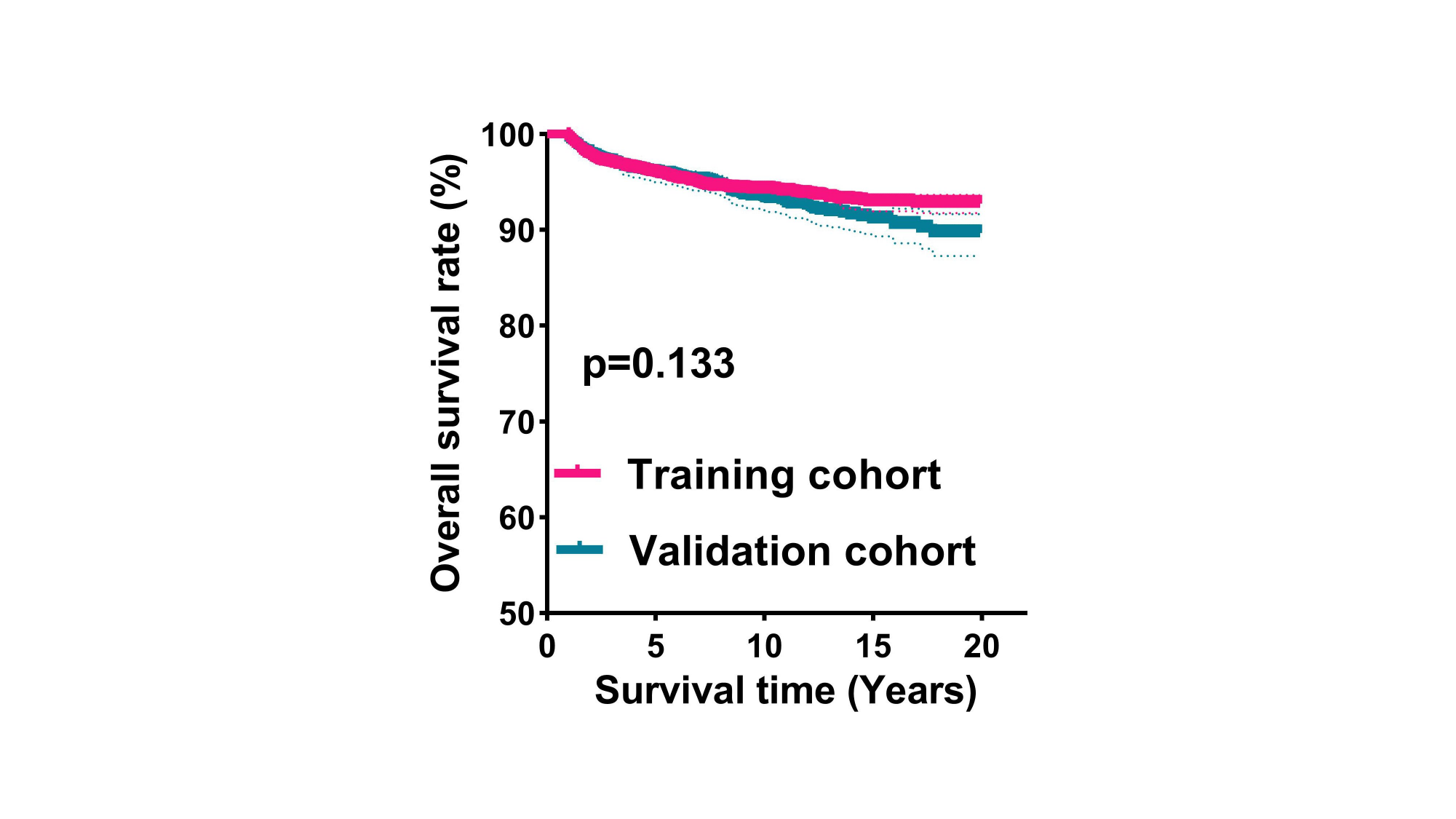

Supplement: Supplementary file 1 [file medi-104-e43781-s001.pptx]
